# *Bacillus anthracis* S-layer protein BslA binds to extracellular matrix by interacting with laminin

**DOI:** 10.1186/s12866-016-0802-8

**Published:** 2016-08-11

**Authors:** Yanchun Wang, Ying Wei, Shengling Yuan, Haoxia Tao, Jie Dong, Zhaoshan Zhang, Wei Tian, Chunjie Liu

**Affiliations:** 1 State Key Laboratory of Pathogens and Biosecurity, Beijing Institute of Biotechnology, Beijng, 100071 China; 2 School of Life Science and Biopharmaceutics, Shenyang Pharmaceutical University, Shenyang, 110016 China

**Keywords:** *Bacillus anthracis*, BslA protein, Laminin, Extracellular matrix, Microbial Surface Components Recognizing Adhesive Matrix Molecules (MSCRAMM)

## Abstract

**Background:**

The *Bacillus anthracis* S-layer protein, BslA, plays a crucial role in mammalian infection. BslA is required to mediate adherence between host cells and vegetative forms of bacteria and this interaction promotes target organs adherence and blood–brain barrier (BBB) penetration in vivo. This study attempts to identify the potential eukaryotic ligand(s) for *B. anthracis* BslA protein.

**Results:**

Biochemical approaches have indicated that the putative host cell ligand(s) for BslA is a surface protein, which is independent of the sugar components for binding to Bs1A. A ligand screening using blot overlays, far Western blots and mass spectrometry analyses revealed that BslA binds to mammalian laminin. ELISA based solid-phase binding assays and surface plasmon resonance assays demonstrated that there were high affinity interactions between BslA_(260–652)_ and laminin. The SPR results also revealed the dissociation constants values of 3.172 × 10^−9^M for the binding of BslA_(260–652)_ to laminin.

**Conclusions:**

These data demonstrated that laminin is a ligand for BslA.

**Electronic supplementary material:**

The online version of this article (doi:10.1186/s12866-016-0802-8) contains supplementary material, which is available to authorized users.

## Background

The key to bacterial infections of host tissues is the establishment of a stable association between the bacterium and host surface structures. This process also is essential for many bacteria for withstanding cellular mechanical cleansing processes and to ultimately allow invasion [1]. To initially adhere to host cells, bacteria possess various surface associated molecules that mediate adherence of the bacteria to the target. Most microbes have specific adhesins, which can bind to specific ligands on the surface of the host cell [2]. Bacterial adhesins bind to host cell ligands via a very specific process, and the specificity of this match determines the range of host cells susceptible to infection by a particular strain of pathogen [3]. One major class of bacterial adhesins consists of proteins that covalently anchor to cell peptidoglycans. These proteins specifically attach to extracellular matrix (ECM) components and are collectively termed microbial surface component recognizing adhesive matrix molecules (MSCRAMMs) [3–5]. MSCRAMMs mediate the initial attachment of bacteria to host tissue, providing a critical step to establish infection. The interaction between MSCRAMM and ECM includes a typical receptor–ligand interaction in which the MSCRAMM serves as the receptor. For several pathogenic microbes, MSCRAMMs play a very important role in their pathogenesis [3, 6, 7]. Recently, anti-adherence strategies for the prevention and treatment of infectious diseases have been considered as an alternative to antibiotics [8, 9]. An increasing number of researchers are focusing on studying the MSCRAMMs and the interaction mechanism of them and related host ligands [6, 10–13].

*Bacillus anthracis*, a Gram-positive, non-motile, rod-shaped, spore-forming bacterium, can cause fatal anthrax, a zoonotic disease [14]. Similar to other bacteria, adherence to host organs also plays a very important role in *B. anthracis* infection. In order to fulfill the ultimate invasion process, bacteria must adhere to some specific tissues and avoid to be eliminated. These interactions first occur between the exosporium (such as BclA) and host [15]. After germination, the surface proteins of the vegetative bacterium must perform a role in adherence to host tissues [16]. To date, two cell-wall anchored collagen-binding proteins (BA0871 and BA5258) have been identified in *B. anthracis*, but this result has not been verified in an appropriate animal model and direct in vivo evidence has also not been used [17]. Agarwal et al. reported that the surface protein α-enolase of *B. anthracis* display laminin-binding activity in vitro and may contribute to invasive potential of *B. anthracis* [18]. However, no evidence has been provided that this protein is an adhesin. Moreover, the capsule and S-layer may baffle these cell-wall anchored proteins to approach their ligands located on host cells [17]. In addition, *B. anthracis* S-layer protein A (BslA) was recently recognized as an adhesin, expressed under host-like conditions, which mediated adherence of vegetative bacteria to various human tissues [19–21].

Prior studies have shown that BslA is necessary and sufficient for adherence of the *B. anthracis* Ames strain to host cells despite the presence of capsule [21]. Guinea pigs infected with a *bslA* mutant strain showed minimal end organ infection, but the animals infected with wild strain displayed disseminated infection [21]. Moreover, BslA-mediated adherence in human endothelial cells is regulated by *B. anthracis* secreted multifunctional metalloprotease, InhA, through promoting BslA degradation. Regulating BslA-mediated adherence, according to the cell phase in the host, enhanced the opportunity to bind to epithelial/endothelial cells and move to target organs for widespread dissemination [22]. Although BslA was the first-identified *B. anthracis* surface-associated adherence that promotes target organs adherence and blood–brain barrier (BBB) penetration in vivo, the host molecule(s) that interacts with BslA protein has (have) not been discovered. In addition, little is known regarding this process. A better understanding of *B. anthracis* adherence mediated by BslA protein at the molecular level is warranted.

To determine the potential eukaryotic ligand(s) for *B. anthracis* BslA protein, we have used various approaches to examine the BslA-mediated interactions between recombinant proteins and host cells. In this study we confirmed that BslA binds to the extracellular matrix by interacting with the laminin, and plays a role as MSCRAMM in *B. anthracis* pathogenesis.

## Results

### Expression, purification and characterization of recombinant proteins

**Fig. 1:**
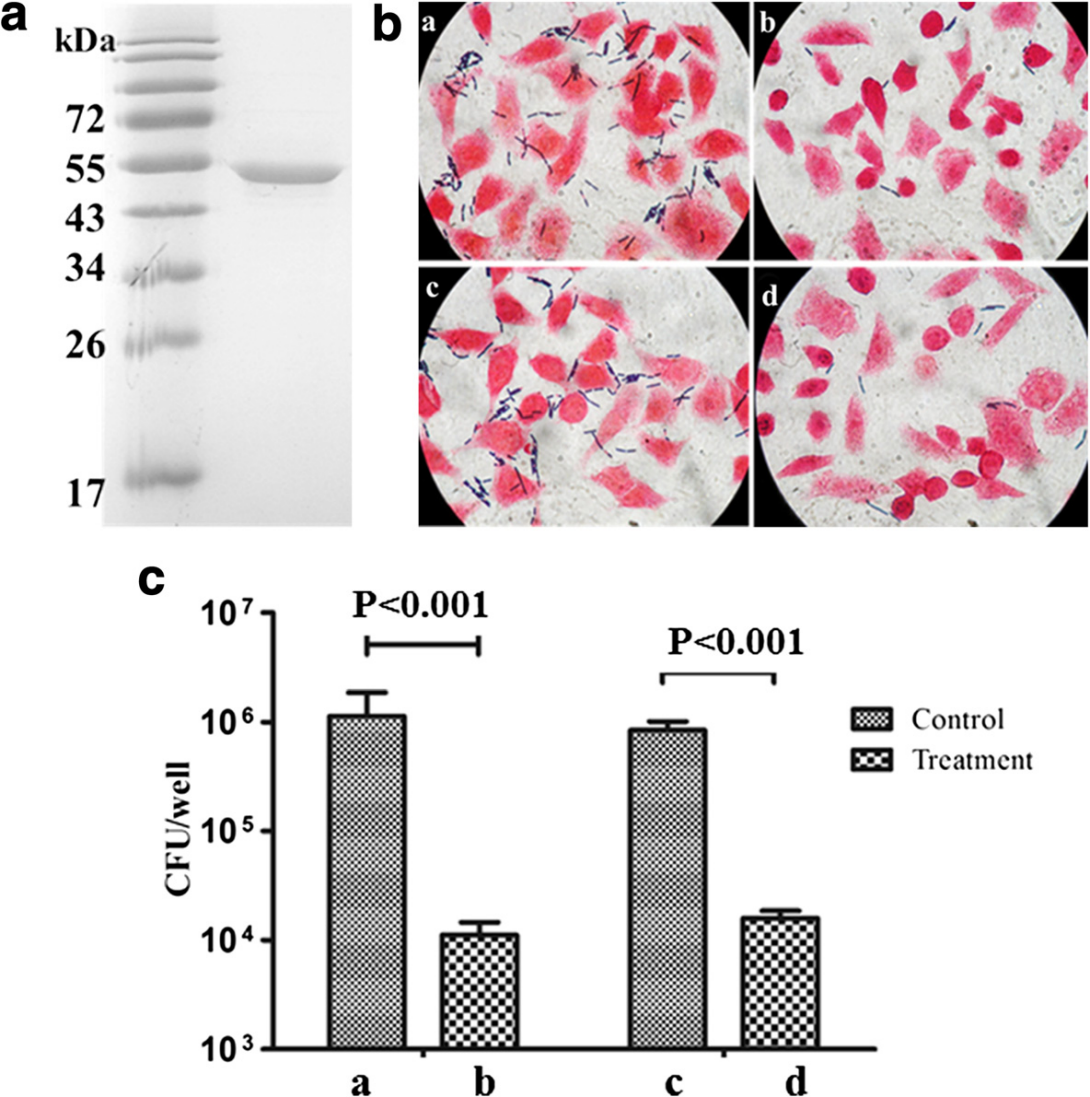
Purification, and characterization of recombinant proteins. **a** Coomassie-stained SDS-PAGE of purified BslA_(260–652)_. **b** Gram stain analysis of the function of BslA_(260–652)_ in adherence of A16R to HeLa cells. a: pretreatment of *B. anthracis* A16R vegetative cell with naïve rabbit sera; b: pretreatment with anti-BslA_(260–652)_ sera; c: pretreatment of HeLa cells with PBS; d: pretreatment with purified BslA_(260–652)_. **c** Histogram of *B. anthracis* colony-forming units (cfu) extracted by lysis of host cells following infections pictured in (**b**). Error bars indicate one SEM. *P*-values were calculated using a two-tailed Student’s *t*-test

To produce soluble rBslA, the truncated protein BslA_(260–652)_ were expressed in *E.coli*BL21(DE3) and purified using Ni Resin. The purity of the recombinant protein was confirmed by SDS-PAGE analysis (Fig. 1a). The purified BslA_(260–652)_ displayed a typical adhesin-like function. Either the anti-BslA_(260–652)_ serum or the BslA_(260–652)_ protein could inhibit *B. anthracis* A16R’s HeLa adherence (Fig. 1b and c). It appears that BslA interacted directly with ligands on the surface of target cells.

**Fig. 2:**
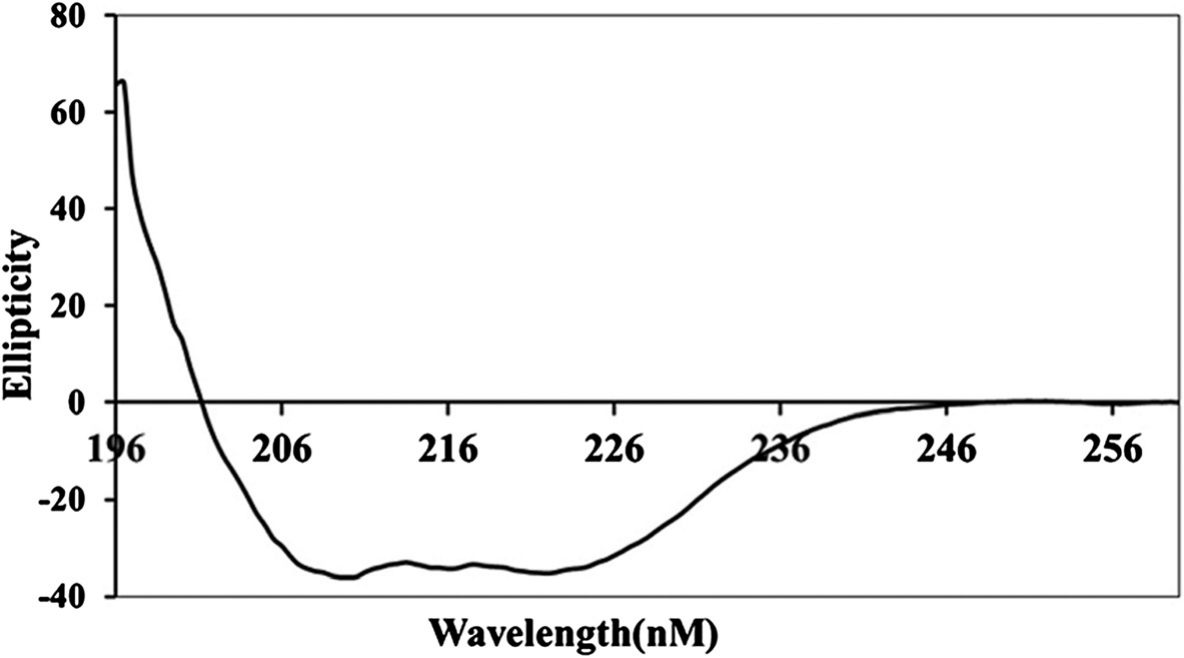
Circulardichroism spectroscopy. The processed spectra were fitted using the computer program CDNN. The spectrum shows that BslA_(260–652)_ is mainly constituted of α-helices (99.9 %, 195–200 nm). The far-UV CD spectrum is presented as a mean of five scans

The secondary structure of BslA_(260–652)_ was analyzed using CD spectroscopy and the NPS@ web server. As shown in Fig. 2, CD spectrum of BslA_(260–652)_ shows the minima at 210 and 222 nm, which is characteristic of α-helical secondary structure content. This suggests a predominance of this structure in the recombinant protein. The percentage of α-helices as predicted by K2D3 is 77.29. The amino acid sequence analysis of BslA_(260–652)_ using NPS@ supported the results of the CD spectrum analysis (Table 1).

**Table 1:** The consensus secondary structure prediction of BslA_(260-652)_ using the NPS@ server^a^

	Prediction method				
	DSC	GOR3	PHD	SOPM	Sec.Cons.
Alpha helix	84.48 %	87.79 %	82.95 %	82.44 %	82.95 %
Beta bridge	0	0	0	0	0
Beta turn	0	0	0	4.83	0
Random coil	13.99 %	8.91 %	15.52 %	10.94 %	14.5 %

^a^Five individual structure prediction algorithms were selected from this analysis, and the consensus prediction is listed on the bottom line

### The ligand(s) of BslA is a protein

**Fig. 3:**
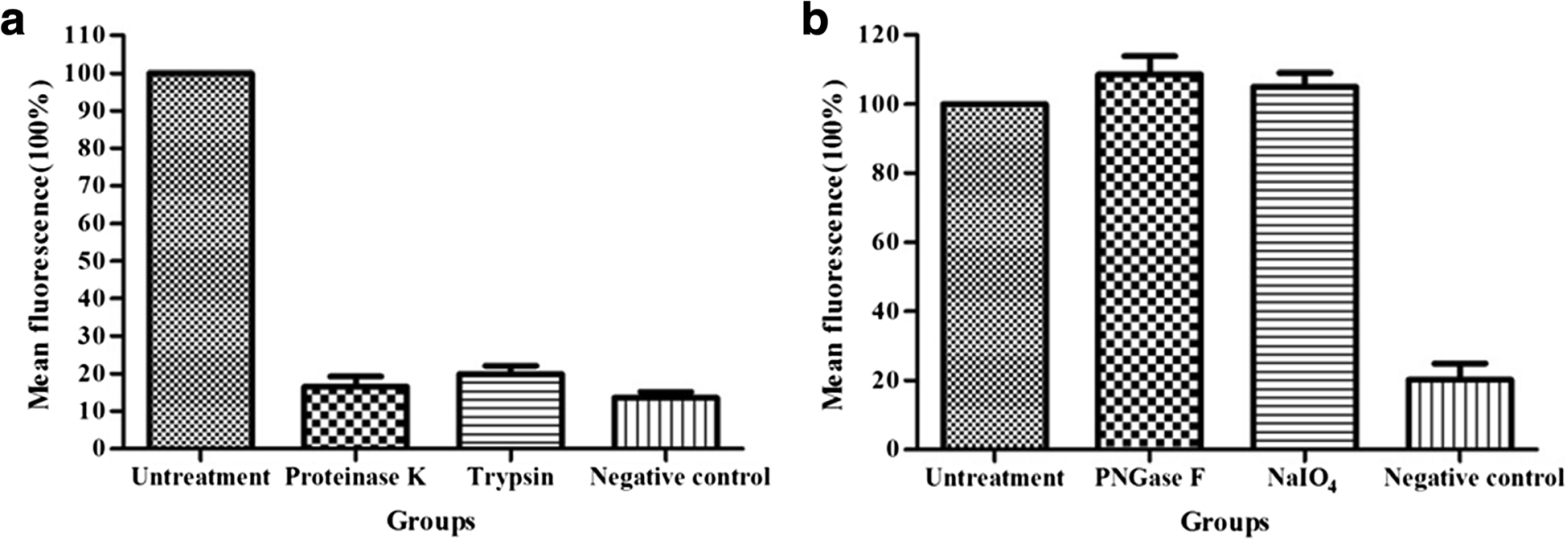
The BslA ligand is a protein; its sugar components are not involved in binding. HeLa cells were treated biochemically (see Methods) and used for binding studies. Quantification of cell surface-bound BslA_(260–652)_ protein on HeLa cells by flow cytometry. The percentage of mean fluorescence in relation to untreated control cells is shown. **a** Cells treated with protease; **b** cells treated with PNGase F or sodium periodate. Cells without BslA_(260–652)_ protein incubated were used as negative controls

To appraise the biochemical nature of the cell surface ligand of BslA, HeLa cells were pretreated with proteinase or various chemical reagents before adding purified BslA_(260–652)_ protein. The protein binding statuses were tested by flow cytometry and a representative tracing were shown in Fig. 3.

When pretreated with proteinase K or trypsin, the binding of BslA_(260–652)_ protein was completely abolished (Fig. 3a). But cleaved with PNGase F or modified with sodium periodate, there were no effect on BslA_(260–652)_ binding (Fig. 3b). These result suggest that the putative host surface ligand(s) of *B.anthracis* BslA may be a surface protein and the target site could be located on the polypeptide chains.

### BslA binds to extracellular matrix by interacting with laminin

**Fig. 4:**
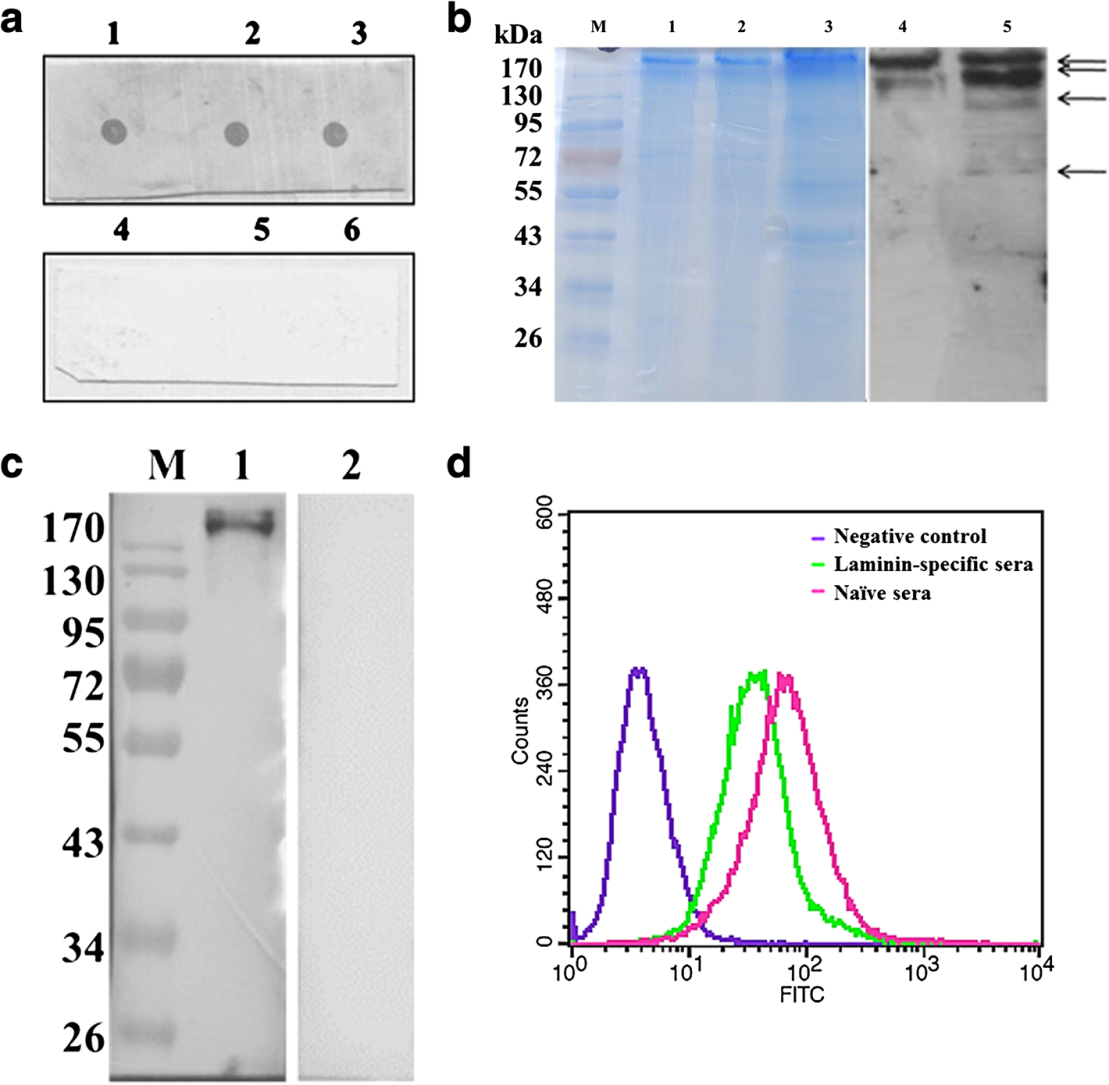
Identification of the interaction between BslA and laminin. **a** Dot Blot (1, 2, 3, ECM incubated with BslA_(260–652),_ triplicate dot; 4,5, 6, ECM incubated with sonicated *E. coli* BL21(DE3) triplicate dot). The result of the overlay suggested that the ligand(s) of BslA are located in the ECM component. **b** Far Western blot. Both non- pretreated ECM (lane 1 and lane 4) and collagenase-digested ECM (lane 2, 3, 5) were used for Far Western blot test. The proteins indicated by arrows were analyzed by MS. **c** Far Western blot using laminin as target protein. Lane 1, both BslA_(260-652)_ and polyclonal BslA antibody was added regularly. Lane 2, only the polyclonal BslA serum was added, negative control. **d** Flow cytometry analysis of the blocking effect of anti-laminin polyclonal antibodies. Cells were preincubated with laminin-specific antibodies (1:50 dilution) or naive rabbit sera for 1 h prior to incubation with BslA_(260–652)_

It had been identified that BslA protein is exposed on the bacterial surface. We examined whether it could adhere to host extracellular matrix proteins, working as a MSCRAMM. To explore the ligand(s) binding of BslA, blot overlay and Far Western blotting were performed using ECM proteins as immobilized targets. As shown in Fig. 4a and b, the BslA_(260–652)_ interacted well with ECM proteins.

To identify the ligand(s) of BslA, the collagenase digested ECM proteins were examined by SDS-PAGE and BslA_(260–652)_ Far-Western blotting (Fig. 4b). Four protein spots were isolated and subjected to ESI-QUAD-TOF-MS analysis after tryptic digestion. The result indicated that peptides belonged to the laminin and could be found in all four protein fragments (Fig. 4b and Additional file 1: Table S1). A second Far Western blot was performed using mice laminin as a target ligand for the purpose of identifying a direct interaction between BslA and laminin (Fig. 4c).

To further confirm that BslA protein bound to laminin on the cell surfaces, BslA_(260–652)_ protein was incubated with COS-7 cells, which had been preincubated with anti-laminin antibodies. When measured by flow cytometry, anti-laminin antibodies inhibited the adherence of BslA_(260–652)_ (Fig. 4d). Thus, BslA appears to function as a *B. anthracis* adhesin that interacts directly with laminin on the surface of target cells.

**Fig. 5:**
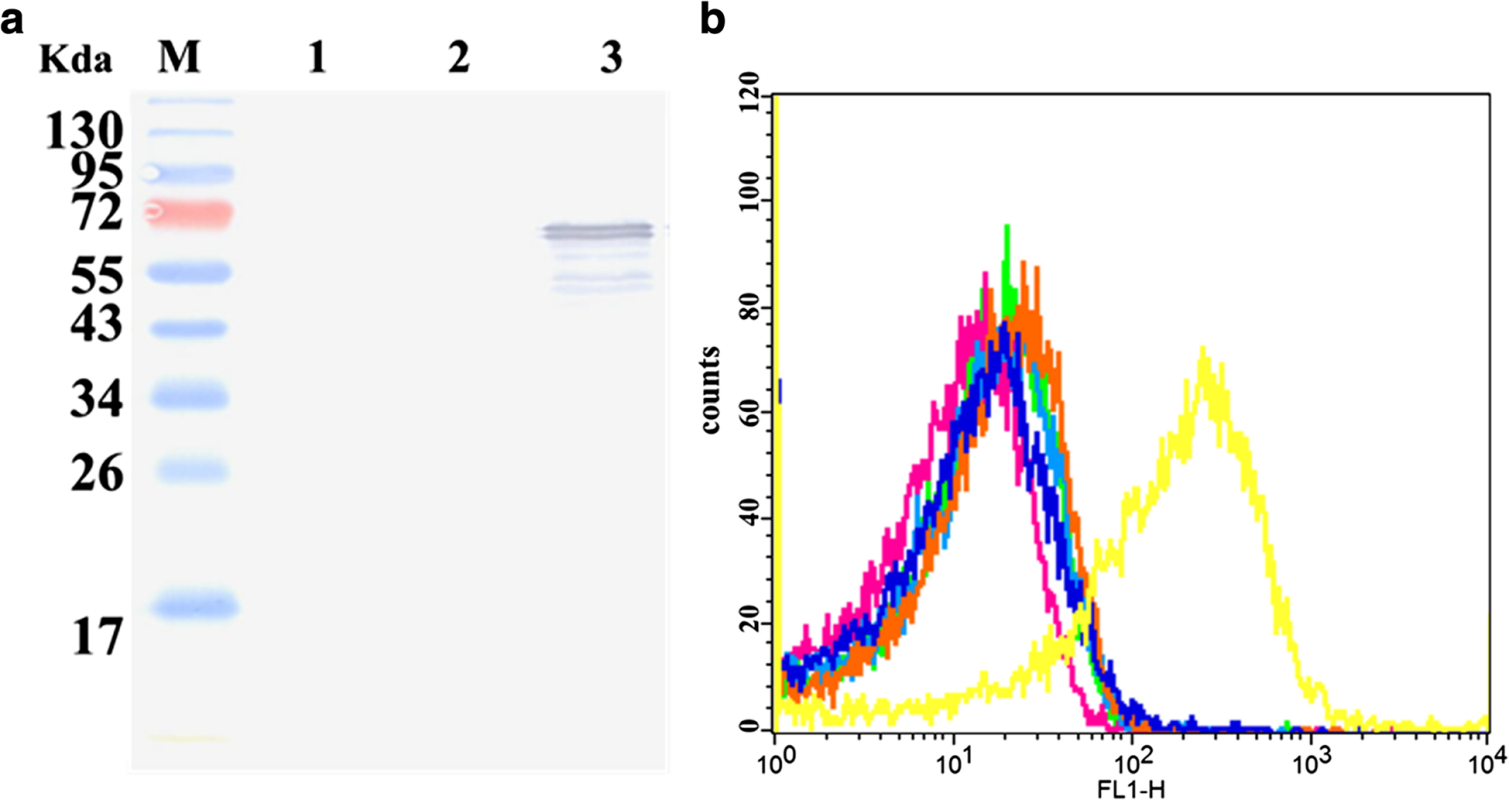
Analysis of laminin binding to *B.anthracis* AP422 expressing BslA. **a** Western blot analysis of the expression of BslA. M, protein maker; lane 1, AP422; lane 2, AP422(pDG148); lane 3,AP422(bslA). **b** Flow cytometry analysis of laminin binding to *B. anthracis* AP422 expressing BslA. All Bacteria were incubated with or without laminin. Each curve represents analysis of 30,000 bacteria

To confirm the binding of laminin to BslA on the *B.anthracis* S-layer, recombinant strain expressed BslA was constructed. Western blot analysis indicated that the BslA expressed well in *B.anthracis* AP422 strain (Fig. 5a). Then the laminin binding was further investigated by flow cytometry according the protocol described in methods.

As shown in Fig. 5b, the AP422 strain (orange line) and AP422(pDG148) (blue line) strain showed a little fluorescence shift following incubation with laminin; however, the AP422(pbslA) strain (yellow line) showed very prominent fluorescence. This result also indicated that laminin bound to *B.anthracis* by interaction with BslA protein.

### BslA_(260–652)_ binds laminin with high affinity

**Fig. 6:**
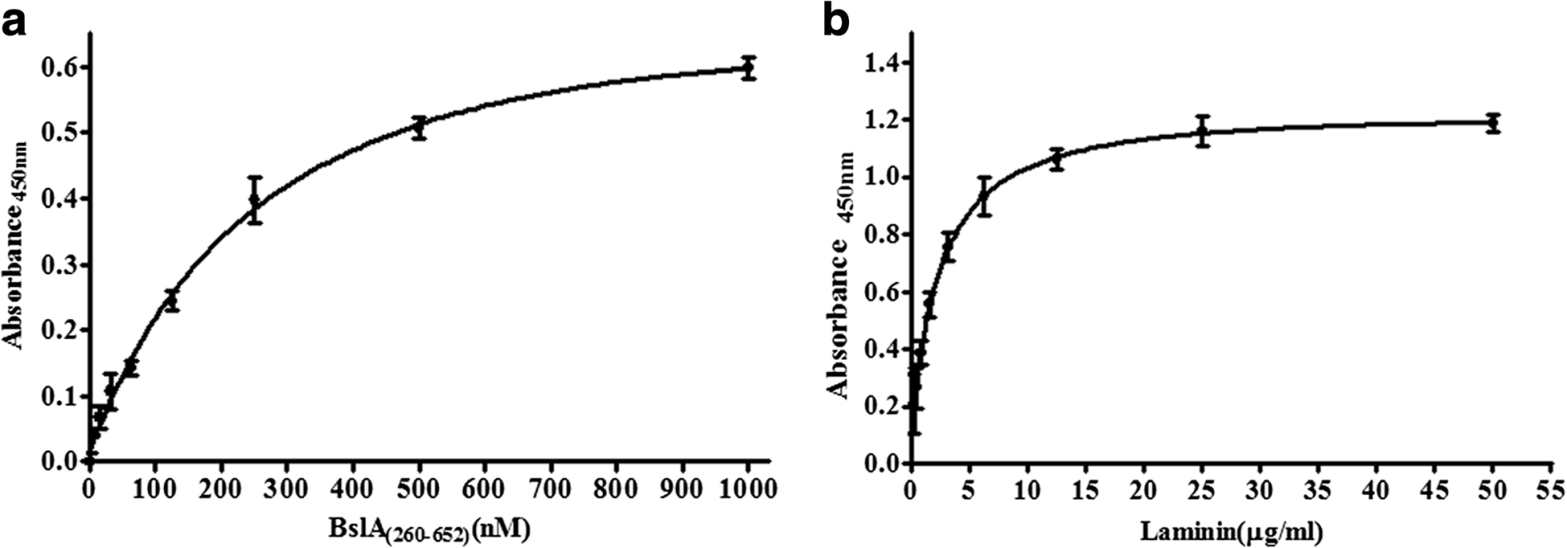
Analysis of the interaction between BslA_(260–652)_ and laminin. **a** ELISA analysis of the binding of different concentrations of BslA_(260–652)_ to immobilized laminin. **b** ELISA analysis of the binding of different concentrations of laminin to immobilized BslA_(260–652)_ with and without the presence of an excess of BslA_(260–652)_ in solution. Responses at equilibrium of the ELISA curves were fit to a one-site binding (hyperbola) isotherm (GraphPad Prism 5). All values are the averages of repeat experiments performed in triplicate with the background absorption subtracted. Error bars indicate SEM

To further characterize this interaction between BslA_(260–652)_ and the laminin, solid-phase binding assays were tested. The results showed that binding of BslA_(260–652)_ to laminin could be fitted to a one-binding-site hyperbola (Fig. 6a). When increasing concentrations of the recombinant protein (0–1000 nM) were allowed to adhere to an immobilized laminin (1 μg/well), a dose-dependent binding was observed. ELISA analysis of the binding of different concentrations of laminin to immobilized BslA_(260–652)_ also indicated that there is a specific interaction between BslA and laminin (Fig. 6b).

**Fig. 7:**
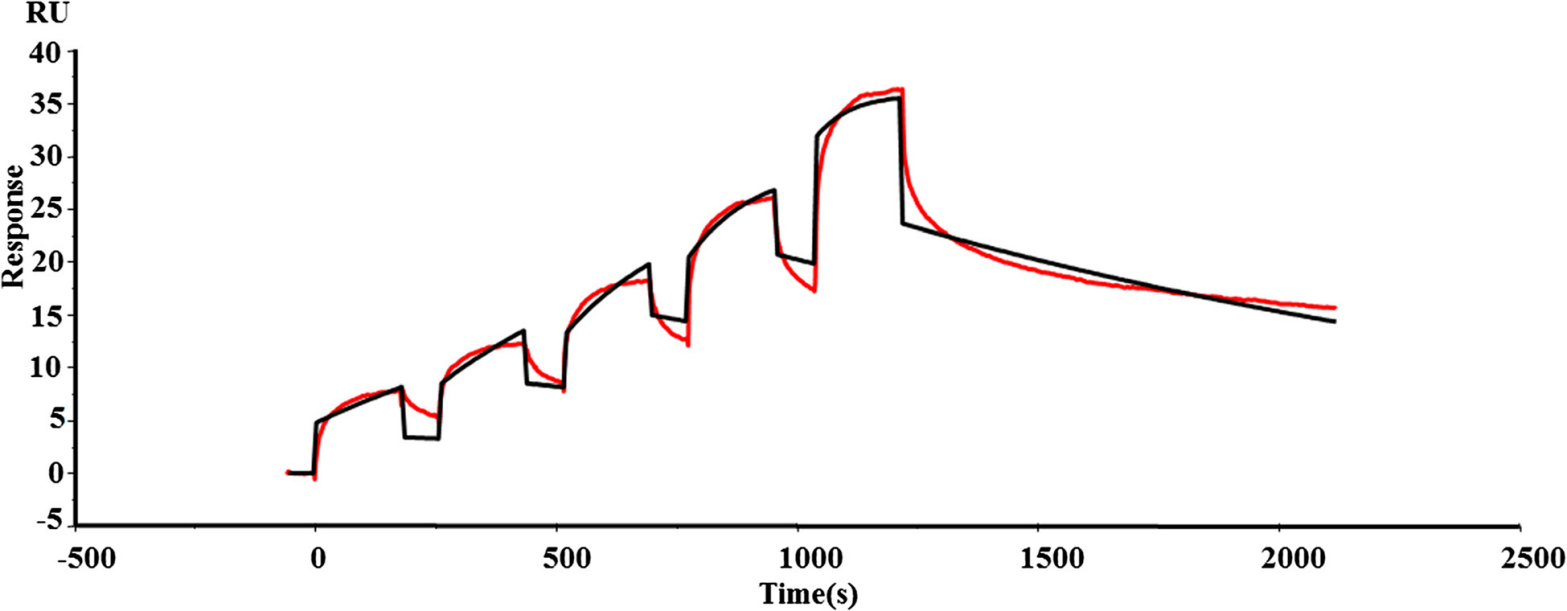
Surface plasmon resonance analysis of BslA_(260–652)_ binding to laminin. Two-fold linear dilution series of BslA_(260–652)_ (4.938, 9.875, 19.75, 39.5 and 79 nM) were injected over the laminin surface (2000 response units) on a BIAcore sensor chip using single-cycle kinetics. Red line contrasts the measured data from the simulated fits (*black line*). Data are representative of two independent experiments

The dissociation constant of the BslA_(260–652)_-laminin complex was determined using SPR (Fig. 7). The *ka*, *kd* and *K*_*D*_ values of BslA_(260-652)_ binding to the laminin chip were calculated. BslA_(260-652)_ possessed a high affinity for laminin(*K*_*D*_ = 3.172 × 0^−9^M, *ka =* 1.749 × 10^5^ M^−1^s^−1^ and *kd =* 5.547 × 10^−4^s^−1^).

## Discussion

Adherence of microorganisms to host surfaces is a critical step in the initiation of infection and is often mediated by surface proteins or adhesins. These processes are commonly mediated by MSCRAMMs, which recognize and adhere to particular host factors, such as fibronectin, collagens, laminin, vitronectin and elastin [4]. Therefore, the search for and identification of MSCRAMMs and their ligands is considered very important in bacterial pathogenesis research. Kern et al observed that BslA truncations lacking amino acids 1–260 can bind directly to fibroblasts BJ-1 and play a key role in bacterial adherence [19], but they did not identify the ligand(s) of this important protein. In this study, using BslA_(260–652)_ as a research tool, we determined that BslA appears to be a characteristic MSCRAMM that interacts with host laminin.

BslA protein is necessary and sufficient for adherence of *B. anthracis* to host cells and very important for anthrax pathogenesis [19, 21]. Mediated by BslA, vegetative forms of *B.anthracis* (both nonencapsulated and encapsulated strains) adhere to host organs, such as liver, kidney and spleen, and permit invasion [19, 21]. The interactions between bacterial adhesins and laminin of host target cells play a key role in several other bacterial pathogens such as that of meningitis [23, 24]. The major role of epithelial laminin is to anchor cells to the basal membrane. Bacterial penetration into the cerebrospinal fluid requires the passage of bacteria through basement membranes, and the interaction of bacterial surface proteins with laminin could be important in this context [25]. BslA-mediated attachment may also contribute to the ability of *B. anthracis* to disrupt the BBB, a key step in anthrax meningitis pathogenesis [20]. We speculate that the interaction between BslA and laminin is a possible reason for BslA protein being very important in anthrax meningitis.

Ebrahimi et al observed that BslA contributed to BBB degradation by disrupting the endothelial tight junctional protein zonulaoccludens (ZO)-1 [20]. As an adhesin, BslA has no protease activity and cannot degrade ZO-1 directly suggesting that other proteases may cause proteolysis. It has been established that *B.anthracis*-secreted metalloprotease InhA disrupts the BBB through proteolytic attack on ZO-1 [26]. InhA also regulates BslA levels at a post-translational level through direct degradation of this protein. InhA serves as a negative regulator of adherence and invasion of B. *anthracis* as a result of this function [22]. At the same time, Nrp599, another secreted neutral metalloprotease of *B. anthracis*, also directly digests host laminin [27]. Therefore, we speculate that the interaction between BslA and laminin was strongly regulated by bacteria. It may be very important to the dissemination of *B. anthracis* during infection occurrence.

For several pathogenic Gram-positive bacteria, laminin is an important ligand molecule of MCSRAMMs [28–31]. Our study also focused on it and identified a corroborative interaction between BslA and laminin. The possibility that there are other ECM molecules interacting with BslA still needs to be explored as the next step. There are number of bacterial adherence molecules with more than one host molecule ligand interactions [32].

## Conclusions

In conclusion, we report here a novel potential ligand (laminin) for BslA, which may help elucidate the important molecular mechanisms involved in the adherence of *B.anthracis* to host organs.

## Methods

### Bacterial strains, culture cells and culture conditions

All bacterial strains used in the present study and their relevant characteristics are listed in Additional file 2: Table S2. All the used *B. anthracis* strains were derivatives of the Chinese vaccine strain A16R. Bacteria were aerobically grown at 37 °C. *Escherichia coli* strains were grown in Luria–Bertani (LB) broth and used as hosts for plasmid cloning and recombinant protein expression. *B. anthracis* strains were grown in brain heart infusion medium with 0.5 % glycerol (BHIG; BD, USA) or LB media. Antibiotics (Merck, Germany) were added to the media when appropriate to the following final concentrations: 100 μg/mL ampicillin for *E. coli*; 50 and 25 μg/mL kanamycin (kan) for *E. coli* and *B. anthracis*, respectively.

HeLa human cervix carcinoma or COS-7 cells were cultured in RPMI-1640 medium supplemented with 2 mM L-glutamine and 10 % FCS in a 5 % CO_2_ atmosphere at 37 °C.

### Recombinant proteins and rabbit-anti-BslA antibodies

Total chromosomal DNA from *B. anthracis*A16R was used as a template to amplify *bslA* truncation using the primers (BslA_(260-652)_ F: 5′-CGGGATCCGAAGAATTGAATCAAAAGTT-3′, BslA_(260-652)_ R: 5′-CCGCTCGAGACTGTTTGGTATTCTAAGTTT-3′). To obtain the recombinant BslA_(260–652)_ protein of *B. anthracis* and prepare its antibody used for the adherence activity studies, the fragment encoding BslA_(260–652)_ was cloned into the pET28a(+) plasmid and induced to express recombinant protein in *E. coli* Rosetta (DE3) by IPTG. The expressed recombinant protein was purified by a column packed with ProBond™ Purification System (Life Technologies) according to the manufacturer’s instructions. Purified protein was used as the antigen to immunize rabbits three times to raise polyclonal antibodies. IgG was purified using protein G-Sepharose (GE Healthcare) affinity chromatography.

### Circular dichroism (CD) spectroscopy

CD spectroscopy measurements were performed in a Chirascan CD Spectrometer (UK Applied Photophysics). The purified recombinant protein was dialyzed using 10 mM sodium phosphate buffer at a concentration of 0.2 mg/ml. Far-UV (196–260 nm) spectra were acquired using a 1-mm-path-length cell at 0.5 nm intervals. Five scans were accumulated and the mean value determined. For the analysis of the secondary structure, these data were analyzed using the online servers K2D3 [33]. Concurrently, the secondary structure prediction also was executed using the Network Protein Sequence Analysis (NPS@site at https://npsa-prabi.ibcp.fr/cgi-bin/npsa_automat.pl?page=/NPSA/npsa_seccons.html) [34].

### BslA_(260–652)_ adherence bioactive assays

Replenished *B. anthracis* A16R cultures, grown to an OD_600_ of 0.5, were inoculated into 12-well tissue culture plates containing monolayers of HeLa cells. Infected monolayers were centrifuged at 600 × g for 5 min to synchronize infection, and then incubated at 37 °C in 5 % CO_2_, 100 % humidity for 4 h. Cells were washed five times with PBS. Standard Gram staining was performed and bacteria were removed from the wells by adding 100 μl of 0.25 % trypsin. Serial dilutions were plated in triplicate on LB agar and grown at 37 °C overnight.

For BslA-specific antiserum inhibition experiments, cultures of *B. anthracis* were preincubated with 2 % BslA-specific or naive rabbit sera for 1 h at 4 °C prior to infection. For protein inhibition experiments, cells were preincubated with purified BslA_(260–652)_ protein (25 μg of protein/1 × 10^6^ cells) for 1 h at 37 °C in FCS free medium and washed twice before infection.

### Biochemical treatment of cells

HeLa cells were grown to 80–90 % confluency and washed with PBS, then detached from the plate with 0.04 % EDTA (10 mM in PBS). Cells were washed and resuspended in approximately 1 × 10^7^ cells/ml in ice cold PBS. For protease treatment, the cells were incubated with 40 μg/ml trypsin or 6 μg/ml proteinase K for 30 min at 4 °C. To degrade the carbohydrate structures on cell surfaces, the cells were incubated with peptide-N-glycosidase F (PNGase F) for 1 h at 4 °C or incubated with 30 mM sodium periodate in 50 mM sodium acetate buffer (pH 4.5) for 1 h at room temperature in the dark. After washing three times with PBS and blocking with 3 % FCS in medium, cells were used for flow cytometry analysis experiments as described further.

### Flow cytometry analysis

BslA_(260–652)_ protein was added to the pretreated cells (25 μg of protein/1 × 10^6^ cells) and incubated for 1 h at 4 °C. Cells were washed three times with PBS, and suspended in PBS containing 3 % FCS and BslA_(260–652)_-specific antiserum (1:100, v/v) and incubated on ice for 1.5 h. After washing 3X with PBS, the cells were incubated with FITC-conjugated goat anti-rabbit IgG (1:1000, v/v; Abcam) on ice for 1 h. Then the cells were stained with 0.5 mg/ml of 7-AAD (KeyGen Biotech) for 10 min and the FITC-labeled cells (7-AAD negative, live) were examined with FACSCalibur (BD Biosciences, USA).

### Blot overlay/Far western blotting assay

For blot overlays, the commercial extracellular matrix (ECM, Corning Life Sciences) were blotted onto nitrocellulose membranes and air dried. For the far Western blot, ECM proteins (collagenase-digested or non-pretreated) were separated on 4–12 % SDS-PAGE and transferred to nitrocellulose membranes. All the membranes were blocked in 5 % fat-free milk in PBST for 1 h. Following several washes with PBST, BslA_(260–652)_ protein in PBST/milk (1 μg/ml) was added to the nitrocellulose membranes, incubated for 1 h at 37 °C, and washed several times. Detection was performed using BslA_(260–652)_-specific antiserum and HRP-conjugated goat anti-rabbit IgG. For blot overlay test, the sonicated *E. coli* BL21(DE3) was used as negative control.

### Mass spectrometry analysis

Further identification of the ligand of BslA was conducted using a combination of the Far Western blotting studies and mass spectrometry (MS). To separate the proteins well and fit mass spectrometry analysis, the ECM protein were digested by collagenase and separated on 4–12 % SDS-PAGE. Separated proteins were electrotransferred to nitrocellulose membranes or stained with Coomassie Blue. The far western blotting was used to determine signals of corresponding protein spots located on the colloidal coomassie gel.

Polypeptides cut out from the coomassie gel were analyzed by ESI-QUAD-TOF. The proteins were identified against the NCBI database using the MASCOT program (www.matrixscience.com), where protein scoring of >82 was significant (*p* < 0.05).

### Blocking assays

For ligand antiserum inhibition experiments, COS-7 cells were preincubated with laminin-specific polyclonal antibodies (Merck Millipore, 1:50 dilution) or naive rabbit sera for 1 h prior to incubation with BslA_(260–652)_ at 4 °C. After washing three times with PBS and blocking with 3 % FCS in medium, cells were labeled with BslA_(260–652)_, followed by mice anti-His tag antibodies and FITC-conjugated rabbit anti-mice IgG for flow cytometry analysis experiments.

### Solid-phase binding assays

To determine binding of BslA_(260–652)_ to immobilized laminin, wells of Costar 96-well plates (Corning) were coated overnight with 20 μg/ml EHS laminin in 50 mM Na_2_CO_3_ (pH 9.6) at 4 °C. Plates washed three times with PBS plus 0.5 % Tween-20 (PBST). Wells were blocked for 2 h at 37 °C with Superblock (Thermo Fisher), and then washed three times with PBST. Wells were incubated for 2 h at 37 °C with 100 μl of various concentrations of recombinant BslA_(260–652)_ in PBST (plus 0.1 % BSA) and developed with BslA_(260–652)_-specific rabbit polyclonal antiserum, followed by goat-anti rabbit HRP-IgG. Plates were washed three times with PBST, incubated for 1 h at 37 °C with HRP-conjugated goat anti-rabbit IgG (Jackson), and diluted 1:5000 in PBST-2 % fat-free milk. After washing, 100 μl of TMB substrate solution (TianGen) was added to the wells in the microtiter plate. After 10 min at room temperature, the reaction was interrupted by adding 50 μl of 1 M H_2_SO_4_. Absorbance was read at 450 nm in a Spectra Max 190 ELISA microplate reader (Molecular Devices). A binding curve was generated using Graphpad Prism, version 5.

To determine the binding of laminin to immobilized BslA_(260–652)_, recombinant BslA_(260–652)_ was diluted in carbonate buffer, pH 9.6, to 1 μg/ml, and 50 μl/well was coated on Costar 96-well plates (Corning) overnight. The wells were washed, blocked, probed with 100 μl of soluble EHS laminin (0–100 mg/ml), and developed with rabbit anti-mouse laminin antibody (Merck Millipore), followed by goat-anti rabbit HRP-IgG.

### Surface Plasmon Resonance (SPR)

All surface plasmon resonance (SPR) experiments were performed using a Biacore T200 instrument at 25 °C (GE Healthcare) using the single-cycle kinetics (SCK) experiments. Briefly, The CM-5 chip was activated with a 1:1 mixture of 0.4 M 1-ethyl-3-(3-dimethylaminopropyl) carbodiimide and 0.1 M N-hydroxysuccinimide for 7 min. Laminin (10 μg/mL in 10 mM sodium acetate buffer, pH 4.0) was immobilized on the surface of individual CM5 chips a flow rate of 5 ml/min. Approximately 2000RU of laminin were immobilized. After immobilization, CM5 chips were inactivated with 1 M ethanolamine-HCl. Increasing concentrations of BslA_(260–652)_ in HBS-EP^+^ buffer(10 mM HEPES,150 mM NaCl,3 mM EDTA,0.05 % P20,pH 7.4) were injected at 30 μl/min over both the ligand and reference surfaces for 3 min then allowed them to dissociate for 15 min at 25 °C. Data processing and analysis were performed using BiacoreT200 evaluation software in a 1:1 binding model (GE Healthcare).

### *B.anthracis* AP422 Expressing BslA

PCR reactions were performed with Pfu DNA polymerase (Transgene) using the primers (BslAF: 5′-GAAGCTTAAGGAGGAAGCAGGTATGAAAAAAAGAAAGATAAAAG-3′, BslAR: 5′-CATGCATGCTTAACTGTTTGGTATTCT-3′). The fragment coding BslA was cloned into the shuttle vector pDG148and the ligation products were transformed into *E. c*oli DH5α, and plasmid DNA into *E. coli* SCS100 (dam-, dcm-) and purified (nonmethylated) plasmid DNA was transformed into *B. anthracis* following a previously developed protocol [35].

### Bacterial adherence laminin assays

Replenished *B. anthracis* AP422, AP422(pDG148) and AP422(*bslA*) cells, induced to express BslA protein by IPTG 4 h before harvest, were washed and resuspended in filtered PBS supplemented with 2 % BSA. Laminin (2 μl of a 1 mg/ml solution) was added to microtubes (1.5 ml), and incubated at 37 °C in the dark for 1 h. Following three washes with PBST, bacteria were incubated for 2 h at 37 °C with a 1:100-diluted rabbit anti-mouse laminin antibody in PBS containing 2 % BSA and incubated on ice for 1.5 h. After washing three times with PBS, the cells were incubated with Alexa Fluor 488 -labeled goat anti-rabbit IgG (1:100, v/v) (Life Technologies) on ice for 1 h. The Alexa Fluor 488-labeled cells were examined using FACSCalibur (BD Biosciences). Unlabeled bacteria AP422 were used as negative controls.

### Statistical methods

All the statistical analyses were performed with the GraphPad Prism 5 software. Data are presented as mean value with the standard deviation (±1 S.D.). Statistical significance was tested using Student’s paired *t*-test or One-way ANOVA. Differences were considered significant at *p* < 0.05. The correlation coefficient (R) and *p*-value (two-tailed) were calculated at 95 % confidence interval.

## Abbreviations

7-AAD, 7-Aminoactinomycin D; BBB, blood–brain barrier; BslA, *Bacillus anthracis* S-layer protein A; CD, Circular dichroism; ECM, extracellular matrix; FCS, fetal calf serum; FITC, Fluorescein isothiocyanate; HRP, Horseradish Peroxidase; MS, mass spectrometry; MSCRAMMs, microbial surface components recognizing adhesive matrix molecules; PNGase F, peptide-N-glycosidase F; SEM, Standard Error of Mean, SPR, surface plasmon resonance, TMB, 3,3′,5,5′-Tetramethylbenzidine

## Additional files

Additional file 1: Table S1. The main protein information detected by mass spectrometry. (DOCX 18 kb) Additional file 2: Table S2. Plasmids and strains used in this study. (DOCX 18 kb)

## References

[1] Pizarro-Cerda J, Cossart P (2006). Bacterial adherence and entry into host cells. Cell.

[2] Kline KA, Fälker S, Dahlberg S, Normark S, Henriques-Normark B (2009). Bacterial adhesins in host-microbe interactions. Cell Host Microbe.

[3] Chagnot C, Listrat A, Astruc T, Desvaux M (2012). Bacterial adherence to animal tissues: protein determinants for recognition of extracellular matrix components. Cell Microbiol.

[4] Patti JM, Allen BL, McGavin MJ, Hook M (1994). MSCRAMM-mediated adherence of microorganisms to host tissues. Annu Rev Microbiol.

[5] Singh B, Fleury C, Jalalvand F, Riesbeck K (2012). Human pathogens utilize host extracellular matrix proteins laminin and collagen for adherence and invasion of the host. FEMS Microbiol Rev.

[6] Heilmann C (2011). Adherence mechanisms of staphylococci. Adv Exp Med Biol.

[7] Kang M, Ko YP, Liang X, Ross CL, Liu Q, Murray BE, Hook M (2013). Collagen-binding microbial surface components recognizing adhesive matrix molecule (MSCRAMM) of Gram-positive bacteria inhibit complement activation via the classical pathway. J Biol Chem.

[8] Cozens D, Read RC (2012). Anti-adherence methods as novel therapeutics for bacterial infections. Expert Rev Anti Infect Ther.

[9] Hartmann M, Papavlassopoulos H, Chandrasekaran V, Grabosch C, Beiroth F, Lindhorst TK (2012). Inhibition of bacterial adherence to live human cells: activity and cytotoxicity of synthetic mannosides. FEBS Lett.

[10] Barbu EM, Ganesh VK, Gurusiddappa S, Mackenzie RC, Foster TJ, Sudhof TC (2010). beta-Neurexin is a ligand for the *Staphylococcus aureus* MSCRAMM SdrC. PLoS Pathog.

[11] Otto M (2008). Targeted immunotherapy for staphylococcal infections: focus on anti-MSCRAMM antibodies. BioDrugs.

[12] Schulte T, Lofling J, Mikaelsson C, Kikhney A, Hentrich K, Diamante A (2014). The basic keratin 10-binding domain of the virulence-associated pneumococcal serine-rich protein PsrP adopts a novel MSCRAMM fold. Open Biol.

[13] Wang X, Ge J, Liu B, Hu Y, Yang M (2013). Structures of SdrD from *Staphylococcus aureus* reveal the molecular mechanism of how the cell surface receptors recognize their ligands. Protein Cell.

[14] Koehler TM (2009). *Bacillus anthracis* physiology and genetics. Mol Aspects Med.

[15] Brahmbhatt TN, Janes BK, Stibitz ES, Darnell SC, Sanz P, Rasmussen SB (2007). *Bacillus anthracis* Exosporium Protein BclA Affects Spore Germination, Interaction with Extracellular Matrix Proteins, and Hydrophobicity. Infect Immun.

[16] Steichen CT, Kearney JF, Turnbough CL (2007). Non-uniform assembly of the *Bacillus anthracis* exosporium and a bottle cap model for spore germination and outgrowth. Mol Microbiol.

[17] Xu Y, Liang XW, Chen YH, Koehler TM, Höök M (2004). Identification and biochemical characterization of two novel collagen binding MSCRAMMs of *Bacillus anthracis*. J Biol Chem.

[18] Agarwal S, Kulshreshtha P, Mukku DB, Bhatnagar R (2008). α-Enolase binds to human plasminogen on the surface of *Bacillus anthracis*. Biochim Biophys Acta.

[19] Kern JW, Schneewind O (2008). BslA, a pXO1-encoded adhesin of *Bacillus anthracis*. Mol Microbiol.

[20] Ebrahimi CM, Kern JW, Sheen TR, Ebrahimi-Fardooee MA, van Sorge NM, Schneewind O (2009). Penetration of the blood-brain barrier by *Bacillus anthracis* requires the pXO1-encoded BslA protein. J Bacteriol.

[21] Kern JW, Schneewind O (2010). BslA, the S-layer adhesin of *Bacillus anthracis*, is a virulence factor for anthrax pathogenesis. Mol Microbiol.

[22] Tonry JH, McNichol BA, Ramarao N, Chertow DS, Kim KS, Stibitz S (2012). *Bacillus anthracis* protease InhA regulates BslA-mediated adherence in human endothelial cells. Cell Microbiol.

[23] Kim KS (2010). Acute bacterial meningitis in infants and children. Lancet Infect Dis.

[24] Tenenbaum T, Spellerberg B, Adam R, Vogel M, Kim KS, Schroten H (2007). *Streptococcus agalactiae* invasion of human brain microvascular endothelial cells is promoted by the laminin-binding protein Lmb. Microbes Infect.

[25] Chung MC, Popova TG, Jorgensen SC, Dong L, Chandhoke V, Bailey CL (2008). Degradation of circulating von Willebrand factor and its regulator ADAMTS13 implicates secreted *Bacillus anthracis* metalloproteases in anthrax consumptive coagulopathy. J Biol Chem.

[26] Mukherjee DV, Tonry JH, Kim KS, Ramarao N, Popova TG, Bailey C (2011). Bacillus anthracis protease InhA increases blood-brain barrier permeability and contributes to cerebral hemorrhages. PLoS One.

[27] Chung MC, Jorgensen SC, Tonry JH, Kashanchi F, Bailey C, Popov S (2011). Secreted *Bacillus anthracis* proteases target the host fibrinolytic system. FEMS Immunol Med Microbiol.

[28] Caswell CC, Oliver-Kozup H, Han R, Lukomska E, Lukomski S (2010). Scl1, the multifunctional adhesin of group A Streptococcus, selectively binds cellular fibronectin and laminin, and mediates pathogen internalization by human cells. FEMS Microbiol Lett.

[29] Jiang S, Wessels MR (2014). BsaB, a novel adherence factor of group B Streptococcus. Infect Immun.

[30] Ragunathan P, Spellerberg B, Ponnuraj K (2009). Structure of laminin-binding adhesin (Lmb) from Streptococcus agalactiae. Acta crystallographica. Section D. Biol Crystallogr.

[31] Ragunathan P, Sridaran D, Weigel A, Shabayek S, Spellerberg B, Ponnuraj K (2013). Metal binding is critical for the folding and function of laminin binding protein, Lmb of Streptococcus agalactiae. PloS one.

[32] Hallstrom T, Singh B, Resman F, Blom AM, Morgelin M, Riesbeck K (2011). *Haemophilus influenzae* protein E binds to the extracellular matrix by concurrently interacting with laminin and vitronectin. J Infect Dis.

[33] Louis-Jeune C, Andrade-Navarro MA, Perez-Iratxeta C (2012). Prediction of protein secondary structure from circular dichroism using theoretically derived spectra. Proteins: Struct, Funct, Bioinf.

[34] Combet C, Blanchet C, Geourjon C, Deléage G (2000). NPS@: network protein sequence analysis. Trends Biochem Sci.

[35] Shatalin KY, Neyfakh AA (2005). Efficient gene inactivation in *Bacillus anthracis*. FEMS Microbiol Lett.

