# Supplementary material for: Bacillus anthracis S-layer protein BslA binds to extracellular matrix by interacting with laminin
Source: BMC Microbiol. 2016 Aug 11;16:183. doi: 10.1186/s12866-016-0802-8 (PMC4981971; doi:10.1186/s12866-016-0802-8)
Supplement: Additional file 1: Table S1. — The main protein information detected by mass spectrometry. (DOCX 18 kb) [file 12866_2016_802_MOESM1_ESM.docx]

Additional file 1: Table S1. The main protein information detected by mass spectrometry.

|  | GI no. | Protein name^a^ | Score | Matches | Sequence coverage |
| --- | --- | --- | --- | --- | --- |
| 1 | 114326497 | Laminin subunit beta-1 | 7587 | 184(144) | 42% |
|  | 153791270 | laminin subunit gamma-1 precursor | 6476 | 152(107) | 40% |
|  | 148706391 | Laminin subunit alpha-1 | 2054 | 47(38) | 16% |
|  | 817975 | laminin B2 (AA 1-217) | 554 | 19(13) | 65% |
| 2 | 171543883 | nidogen-1 precursor | 6752 | 164(99) | 50% |
|  | 148704971 | laminin B1 subunit 1 | 2449 | 59(46) | 23% |
|  | 293691 | laminin B2 | 1271 | 29(25) | 18% |
|  | 309420 | laminin, alpha 1 | 891 | 29(21) | 9% |
| 3 | 171543883 | nidogen-1 precursor | 4296 | 104(74) | 44% |
|  | 114326497 | laminin subunit beta-1 | 3390 | 78(64) | 29% |
|  | 153791270 | laminin subunit gamma-1 precursor | 1231 | 35(24) | 17% |
|  | 148706391 | laminin, alpha 1 | 1112 | 40(28) | 11% |
| 4 | 163310765 | serum albumin precursor | 3191 | 102(67) | 67% |
|  | 74143862 | unnamed protein product | 2286 | 57(49) | 53% |
|  | 148706391 | laminin, alpha 1 | 274 | 11(6) | 3% |
|  | 293691 | laminin B2 | 273 | 9(5) | 6% |

a, protein taxonomy: *Mus musculus*
