# Supplementary material for: Bacillus anthracis S-layer protein BslA binds to extracellular matrix by interacting with laminin
Source: BMC Microbiol. 2016 Aug 11;16:183. doi: 10.1186/s12866-016-0802-8 (PMC4981971; doi:10.1186/s12866-016-0802-8)
Supplement: Additional file 2: Table S2. — Plasmids and strains used in this study. (DOCX 18 kb) [file 12866_2016_802_MOESM2_ESM.docx]

| Plasmids and strains | Relevant characteristics | Source or reference |
| --- | --- | --- |
| plasmids |  |  |
| pET28a(+) | Expression vector, Kan^R^ | Merck |
| pET-BslA_(260–652)_ | *bslA*codons 260 to 652 in pET28a(+) | This work |
| pDG148 | Amp^R^, Kan^R^ , *B.anthracis*–*E.coli* shuttle expression vector | [1] |
| pDG-BslA | pDG148 containing bslA | This work |
| *E. coli* |  |  |
| DH5α | High competency cloning strain | Transgene |
| BL21(DE3) | strain used to express recombinant proteins | Transgene |
| SCS110 | *dam*-/*dcm*- strain used to produce unmethylated plasmid DNA | Stratagene |
| *B. anthracis* |  |  |
| A16R | pXO1**^+^**pXO2**^-^**, China vaccine strain. | [2] |
| AP422 | pXO1**^-^**pXO2**^-^**, deriving from A16R | This lab |
| AP422(pDG148) | AP422 expressing pDG148 | This work |
| AP422(pbslA) | AP422 expressing bslA in pDG148 | This work |

Additional file 2: Table S2 Plasmids and strains used in this study

1. Stragier P, Bonamy C, Karmazyn-Campelli C. Processing of a sporulation sigma factor in *Bacillus subtilis*: how morphological structure could control gene expression. Cell. 1988; 52:697−704.
2. Liu XK, Qi XP, Zhu L, Wang DS, Gao ZQ, Deng HJ, et al. Genome sequence of Bacillus anthracis attenuated vaccine strain A16R used for human in China. J Biotechnol, 2015; 210:15–16.
